# A Prototype Skin Substitute, Made of Recycled Marine Collagen, Improves the Skin Regeneration of Sheep

**DOI:** 10.3390/ani11051219

**Published:** 2021-04-23

**Authors:** Luca Melotti, Tiziana Martinello, Anna Perazzi, Ilaria Iacopetti, Cinzia Ferrario, Michela Sugni, Roberta Sacchetto, Marco Patruno

**Affiliations:** 1 Department of Comparative Biomedicine and Food Science, University of Padova, Viale dell’Università 16, Legnaro, 35020 Padova, Italy; luca.melotti@unipd.it (L.M.); roberta.sacchetto@unipd.it (R.S.); 2 Department of Veterinary Medicine, University of Bari, SP. Casamassima Km.3, Valenzano, 70010 Bari, Italy; tiziana.martinello@uniba.it; 3 Department of Animal Medicine, Production and Health, University of Padova, Viale dell’Università 16, Legnaro, 35020 Padova, Italy; anna.perazzi@unipd.it; 4 Department of Environmental Science and Policy, University of Milan, Via Celoria, 2, 20133 Milan, Italy; cinzia89.ferrario@gmail.com; 5 Center for Complexity and Biosystems, Department of Physics, University of Milan, Via Celoria, 16, 20133 Milan, Italy

**Keywords:** marine collagen, wound healing, regenerative medicine, innovative therapies, skin substitute, sea urchin, biomaterials, tissue engineering, circular economy, 3D scaffolds

## Abstract

**Simple Summary:**

Marine ecosystems are a huge source of unexplored “blue” materials for different applications. The edible part of sea urchin is limited, and the vast majority of the product ends up as waste. Our studies intend to fully recycle wastes from the food industry and reconvert them in high added-value products, as innovative biocompatible skin substitutes for tissue regeneration. The aim of the present work is to apply the pioneering skin substitute in in vivo experimental wounds to test its regenerative potential and compare it, in a future study, to the available commercial membranes produced with collagen of bovine, porcine, and equine origin. Results are encouraging since the skin substitute made with marine collagen reduced inflammation, promoted the deposition of granulation tissue, and enhanced a proper re-epithelialization with the adequate development of skin appendages. In summary, our findings might be of great interest for processing industries and biotech companies which transform waste materials in high-valuable and innovative products for Veterinary advanced applications.

**Abstract:**

Skin wound healing is a complex and dynamic process that aims to restore lesioned tissues. Collagen-based skin substitutes are a promising treatment to promote wound healing by mimicking the native skin structure. Recently, collagen from marine organisms has gained interest as a source for producing biomaterials for skin regenerative strategies. This preliminary study aimed to describe the application of a collagen-based skin-like scaffold (CBSS), manufactured with collagen extracted from sea urchin food waste, to treat experimental skin wounds in a large animal. The wound-healing process was assessed over different time points by the means of clinical, histopathological, and molecular analysis. The CBSS treatment improved wound re-epithelialization along with cell proliferation, gene expression of growth factors (VEGF-A), and development of skin adnexa throughout the healing process. Furthermore, it regulated the gene expression of collagen type I and III, thus enhancing the maturation of the granulation tissue into a mature dermis without any signs of scarring as observed in untreated wounds. The observed results (reduced inflammation, better re-epithelialization, proper development of mature dermis and skin adnexa) suggest that sea urchin-derived CBSS is a promising biomaterial for skin wound healing in a “blue biotechnologies” perspective for animals of Veterinary interest.

## 1. Introduction

Skin lesions are a common event in the veterinary practice, regardless of sex or age, and they may be of different etiology such as trauma, burns, fights, predation, etc. In addition, these injuries can often result in loss of skin function due to extensive scarring or inability of the skin to close the wound (e.g., excessive loss of tissue after tumor resection or bite wounds). Indeed, the treatment of these kind of wounds, which might evolve into chronic or non-healing wounds, is a challenging and expensive issue to manage in the veterinary clinical field of domestic animals [1,2,3]. Wound healing is a complex multi-phase (hemostasis, inflammation, proliferation, and remodeling) process, which is regulated and led by the dynamic interplay of cells, growth factors, and the extracellular matrix (ECM) [4]. Large skin wounds are commonly left open to heal by second intention in veterinary patients like horses or dogs [5,6]. Nonetheless, this common practice has disadvantages such as delayed healing and excessive wound contracture with consequent tissue dysfunction (e.g., a wound on the limb) [7,8]. Nowadays, the application of skin autografts to treat severe non-healing wounds is considered the gold standard [9]. However, this practice has many limitations such as the availability of healthy donor tissue or high pain level for the patients. In addition, graft failure is frequently observed as a consequence of excessive inflammation, infection, or animal locomotion [9,10]. Tissue engineering might offer a solution to this health issue by creating a scaffold that can accelerate wound closure and support the complete regeneration of the skin in terms of function and anatomy, namely skin appendages and anatomical structure.

According to Ferreira and colleagues [11], “skin substitutes are a heterogeneous group of biological and/or synthetic elements that enable the temporary or permanent occlusion of wounds”. To date, many tissue-engineered products have been used to treat skin defects. The ideal skin substitute should resemble the skin structure and architecture in order to stimulate and support wound healing. In general, the aim of researchers is to create an easy-to-handle and resistant skin substitute. Different materials of different origin, such as bovine collagen, polyglycolic acid, or acellular cadaver dermis [12,13,14,15], have been exploited to obtain skin substitutes, which could also contain lipids, fibrin, glycosaminoglycans, and proteoglycans in addition to collagen as its main substituent. Among them, the use of collagen-based biomaterials, being the main protein of the ECM, has been widely investigated for tissue engineering studies and showed promising results. As a matter of fact, the application of these biomaterials for the treatment of skin wounds has been reinforced for their characteristics such as biocompatibility [16], low immunogenicity [17], and bioactive properties [18].

Since, a one-step skin grafting and a split-thickness skin graft approach to cover the wound at the same time is desirable, numerous bi-layer skin substitutes have been developed for the management of full-thickness skin defects, such as Alloderm^®^ [19,20], Integra^®^ [21], Pelnac^®^ [22], and Matriderm^®^ [23,24]. These commercial matrices are all produced with collagen of bovine, porcine, and equine origin. In the last years, alternative collagen sources have been investigated to overcome ethical and economical problems associated with collagen of the aforementioned origin because of risk of diseases transmission (e.g., BSE, bovine spongiform encephalopathy); among these, marine organisms have been proposed as promising sources of collagen [25,26,27]. Nevertheless, the majority of marine-derived collagens (sponges, jellyfish, mollusks, and fish), as well as bovine or swine collagens, are used in their hydrolyzed form, since hydrolysis is a step necessary for an efficient extraction. However, this chemical extraction presents two main limitations. First of all, the collagen-associated molecules, i.e., GAGs, are generally lost during the hydrolysis and they need to be artificially added to reproduce the natural characteristics of the ECM, such as hydration [28]. Moreover, when collagen is re-assembled in vitro to form fibrils [29], it often fails to fully reconstitute the original structure and, therefore, its functional efficiency, providing sub-optimal mechanical features [30]. Echinoderms, in particular sea urchins, are one of the most promising source of collagen among marine invertebrates [31,32]. In fact, they offer the possibility to extract collagen fibrils in their native conformation [31,32] with endogen fibril-associated GAGs [31,33], obtaining a biomaterial resembling the in vivo structural microenvironment. In addition, sea urchin collagen has the remarkable advantage to be extracted from wastes originating from the food industry (restaurants and seafood enterprises), thus following circular economy principles and sustainable approaches [31].

In a previous study [27], we described the production and the characterization of sea urchin (*Paracentrosus lividus*)-derived collagen-based skin-like scaffolds (CBSS) composed by an “epidermis-like” layer and a “dermis-like layer,” i.e., a thin 2D collagen membrane and a sponge-like 3D collagen scaffold, respectively. In that work, we demonstrated that the sea urchin-derived CBSS is a promising biomaterial for biomedical applications because of its in vitro non-cytotoxicity and barrier function against bacteria, dehydration, and protein loss. Moreover, it possesses the advantage to be biodegradable, thus in an in vivo application no further removal operation of the synthetic upper layer will be necessary as observed for example with Integra^®^.

The aim of this current work was to apply this innovative skin substitute in in vivo experimental wounds to test its regenerative potential using a sheep surgical wound model. Wounds were treated with the CBSS and clinical, histopathological, and molecular studies were carried out to assess its effects on the wound healing process. The obtained results provide evidences that CBSS could lead to a reduction of inflammation, promote granulation tissue maturation, and enhance re-epithelialization along with the development of skin appendages.

## 2. Materials and Methods

### 2.1. Animal Model and Ethical Statement

Three female 2-year-old Bergamasca sheep were included in this experimental study. Sheep were allocated in an experimental barn (MAPS Department, University of Padova, Legnaro, Italy) at least two weeks prior to the start of the experimental study for acclimating themselves. In addition, the animals were subjected to clinical, hematological, and parasitological examinations in order to assess their health status before beginning the study. The animals had ad libitum access to water and feed.

The experiment was approved by the Italian Ministry of Health (n°51/2015-PR), in accordance with the Body for the Protection of Animals (OPBA). The number of animals included in the present study was based on a sample size calculation, taking into account the “3Rs” principle [34]. At the end of the experimentation, sheep were transferred to a teaching farm.

### 2.2. Sea Urchin Collagen Extraction, Production of 2D Membranes, and 3D Scaffolds

Fibrillar collagen in its native conformation, i.e., with its typical decoration of glycosaminoglycans (GAGs), was extracted from the sea urchin *Paracentrotus lividus*, specifically from the peristomial membranes obtained from restaurant wastes, as previously described [31,32].

**Figure 1:**
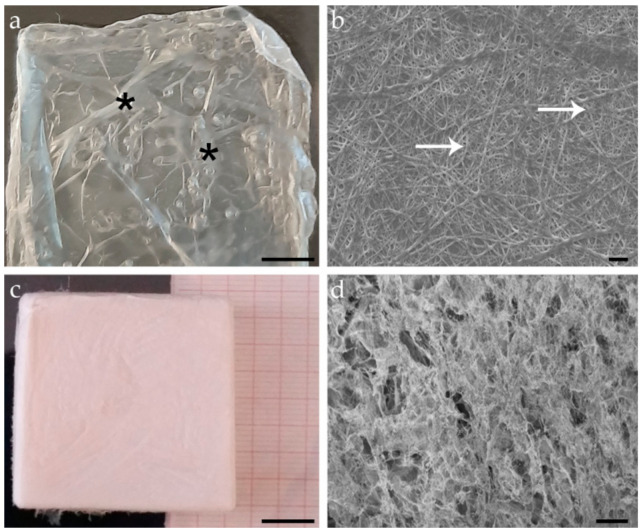
Examples of 2D membranes and 3D scaffolds of sea urchin-derived collagen. (**a**) Top view of a 2D membrane (light microscopy). Asterisks mark macroscopic folds of the thin 2D membrane. (**b**) Micrograph of a 2D membrane where the random distribution of the single collagen fibrils (arrows) in the two-dimensional network is visible (scanning electron microscopy). (**c**) Top view of a 3D scaffold (light microscopy). (**d**) Micrograph of a 3D scaffold where the porous microstructure of the biomaterial is detectable (scanning electron microscopy). Scalebar: (**a**) = 500 µm; (**b**) = 2 µm; (**c**) = 1 cm; (**d**) = 200 µm.

The so obtained suspension of fibrillar collagen in autoclaved filtered distilled water (dH_2_O) was stored at −80 °C until use. After thawing at room temperature (RT), it was used to produce both 2D thin membranes and 3D sponge-like scaffolds following two different procedures, as described in [27,32]. Briefly, 2D membranes (about 4 cm × 4 cm × 10–15 µm) were produced by adding a proper amount of collagen suspension (2 mg/mL in 0.01% TritonX-100 in autoclaved filtered dH_2_O) to rubber silicone molds of the desired size and left to dry at 37 °C overnight. 3D scaffolds (about 4 cm × 4 cm × 1.5 cm) were produced by adding a proper amount of collagen suspension (6 mg/mL) in 6% ethanol in autoclaved filtered dH_2_O to rubber silicone molds, frozen overnight at −80 °C, and lyophilized (Edwards Pirani 1001) overnight. Finally, both 2D membranes and 3D scaffolds were cross-linked and sterilized under a 15 W UV lamp at RT overnight (Figure 1). The so obtained sea urchin-derived collagen biomaterials (CBSS) were stored at −20 °C until use. Immediately before the surgical operation, they were sterilized for 1 h under an UV lamp.

### 2.3. Surgical Procedure and Animal Model

A total of six full-thickness skin defects (16 cm^2^, 4 cm × 4 cm) were created on the back of each sheep under general anesthesia ensuring complete analgesia. The wounds were equidistant (6 cm ipsilteral and 10 cm contralateral) and the distance between them did not affect the healing process or the outcomes of the experiment. The loss of skin tissue was considered significant and the wound edges could not be approximated: wounds will heal by second intention [35]. Five wounds were treated with five different therapies while one was left untreated, and considered as the control wound. The application of the five different therapies and the untreated (control) wound were randomized for each sheep. In the current study, we consider the application of the previously described CBSS comparing it to control wounds. Other treatments, not related to the application of biomaterials, are reported in other original research manuscripts [36].

Before surgery, the animals were treated with an antibiotic (amoxicilline, 15 mg/Kg) and an analgesic therapy (tramadol, 4 mg/Kg) via intramuscular injection. Then, they were premedicated and sedated with medetomidine (0.01 mg/Kg) via intravenous administration. After a proper level of sedation, the animal was placed in sternal decubitus position and the trichotomy of the *thoracolumbar* area was performed for the preparation of the surgical field. Anesthesia was induced by administering propofol (4 mg/Kg). Afterwards to maintain anesthesia, the animal was orotracheally intubated and administered with isoflurane combined with a mixture of oxygen and medical air.

**Figure 2:**
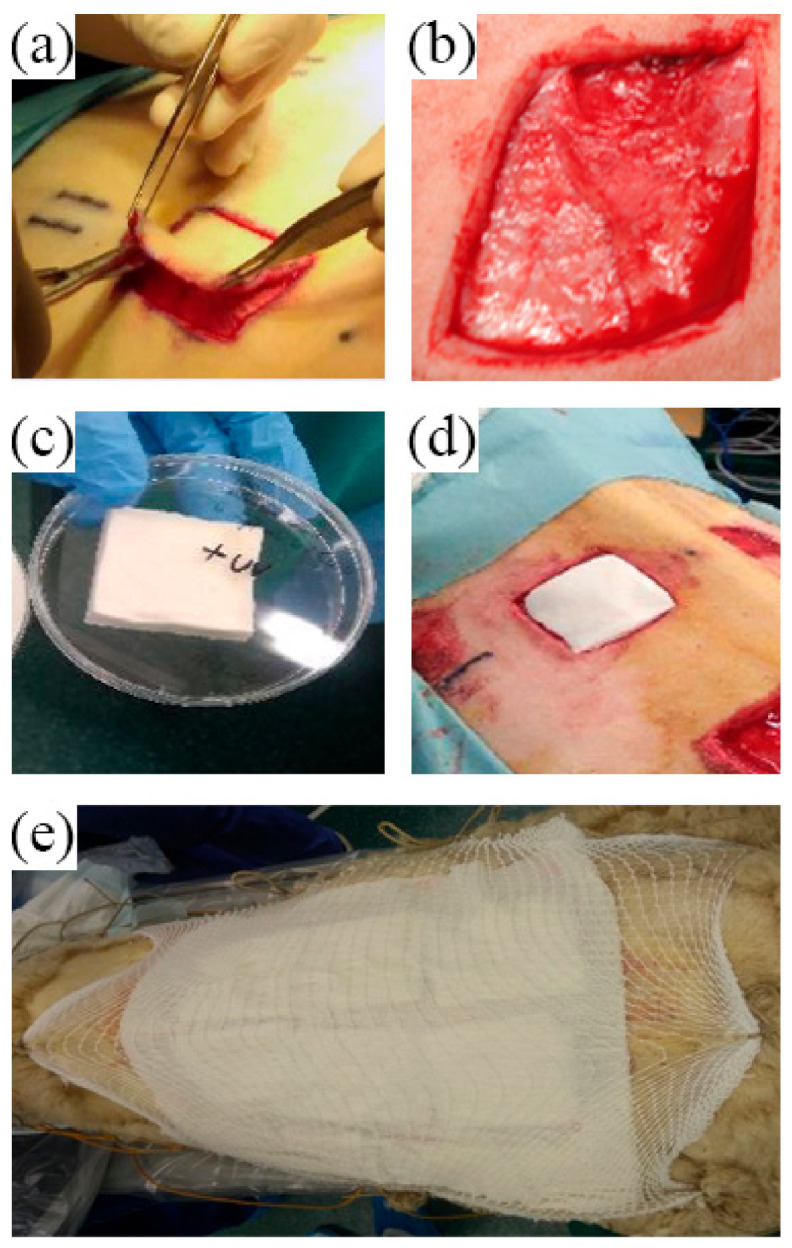
Representative images of the surgery procedure and biomaterial implantation. (**a**) Skin full-thickness removal by using surgical scissors for detaching the dermis from the subcutis; (**b**) macroscopic appearance of the wound after surgery; (**c**) 3D scaffold after UV sterilization and before implantation; (**d**) 3D scaffold implantation in the wound, the biomaterial was directly placed onto the wound bed; (**e**) representative figure of the bandages applied to the back of each sheep after the surgery.

The skin on the back of each sheep was marked using as a guide a sterilized square model (as a template) in order to designate the wound area where to perform the skin surgery. At the same time, this procedure was performed to take into account the skin retraction during wound healing in order to have a representative collection of skin biopsies at different time points. After scrubbing the surgical field with iodine-povidone (10%), six square full-thickness skin wounds (16 cm^2^, 4 × 4 cm) were created on the back of each sheep using the previously marked point as a guide. After skin incision to define the wound borders with a sterile surgical blade, the skin was removed using dissection scissors (Figure 2a,b). Then, one of the wounds was covered with the CBSS: first, the dermal layer (3D porous layer) (Figure 2c) was placed on the wound bed (Figure 2d) followed by the placement of the epidermis-like layer (2D thin membrane) on the top of the dermal one. Afterwards, each wound was medicated and covered with a sterile non-woven gauze. The sterile gauzes were covered with a protective covering bandage and a tubular mesh gauze, which was fixed to the peripheral wool to keep bandages on site (Figure 2e). The covering bandages were changed every three days until day 42. In order to change them, non-woven gauzes were wet with a sterile saline physiological solution to avoid damage to the healing surface.

After surgery, the animals were all housed in an adequate barn to ensure their well-being as gregarious animals. The covering bandage was enough to prevent allogrooming between animals. They received antibiotic therapy (amoxicilline, 150 mg/Kg) for five days and anti-inflammatory therapy (carprofen, 3.5 mg/Kg) for two days. Both therapies were performed by subcutaneous injection.

At 7, 14, 21, and 42 days after surgery skin biopsy were collected for assessing the skin wound healing process and comparing the efficacy of the treatment to control wounds (i.e., physiologically healing wounds). Biopsies collection was performed with the animals properly sedated and treated with medetomidine (0.01 mg/Kg, IV injection) and tramadol (4 mg/Kg, intramuscular injection) for analgesia and pain management. At each time point, two biopsies were collected by means of a 6-mm punch for all lesions. Skin samples were collected in two different and precise position of the wound, opposite to each other and equidistant from the original wound margins (day 0), previously marked on the healthy skin, after shearing the wool. Biopsies were then processed as follows: one for histological analysis (histopathology and immunohistochemistry) and one for gene expression analysis (real time PCR).

### 2.4. Clinical Follow-Up

The macroscopic appearance of each lesion was evaluated at 3, 7, 14, 21, and 42 days after surgery by taking pictures. This operation was performed by the same blinded operator at each time point. Moreover, the percentages of re-epithelialization and wound contraction were measured and calculated using an image processing program (ImageJ^®^).

### 2.5. Histopathological and Immunohistochemical Analysis

A total of 24 biopsies, 6 for each time point (3 for treated and 3 for untreated lesions), were processed for histological analysis. Skin samples were fixed in 10% neutral-buffered formalin (4% formaldehyde) for 24 h. They were then dehydrated by using a gradual dilution of ethanol and embedded in paraffin following standard procedures. After embedding, samples were cut with a microtome (Leica—RM2035, Leica Microsystems, Wetzlar, Germany) into 4-µm thick slices. 

For histopathological evaluation, sections were stained with Harris hematoxylin and eosin (H&E) following the standard protocol. All sections were examined by two blinded independent operators and assessed for different histological parameters: superficial and deep inflammation, immature granulation tissue, and development of skin adnexa. The parameters were scored with a semi-quantitative scale from 0 to 3 (0 absence, 1 mild, 2 moderate, 3 abundant). Data were calculated for each animal and parameter, and presented as relative frequencies. The histopathological evaluation was performed by observation under a light microscope (Olympus Vanox AHBT3, Olympus, Tokyo, Japan). 

In addition, the epidermal thickness index (ETI) was calculated to define the epidermal hypertrophy degree. To do so, the average thickness of the epidermis of the skin samples at 0, 21, and 42 days after surgery was measured in ten randomly selected fields of view for each slide (400× magnification). The ETI was calculated based on an Equation (1) described by Rahmani-Neishaboor and colleagues [37]:

(1) $$\mathrm{ETI}\;=\frac{\mathrm{height}\;\mathrm{of}\;\mathrm{epidermis}\;\mathrm{at}\;\mathrm{day}\;21/42}{\mathrm{height}\;\mathrm{of}\;\mathrm{epidermis}\;\mathrm{at}\;\mathrm{day}\;0}$$

ETI values equivalent to 1 indicate a full healing of the wound without scar formation while values >1 represent a newly formed hypertrophic epidermis.

For immunohistochemistry, samples were immunostained with Ki67 (1:50, Dako, Santa Clara, CA, USA), a nuclear marker for cell proliferation, and alpha smooth muscle actin (α-SMA, 1:1000, Dako, Santa Clara, CA, USA), a marker to detect the presence of myofibroblasts (Table 1), following the manufacturer’s protocol. Briefly, sections were deparaffinized and rehydrated by immerging them in graded ethanol series. In order to avoid false positive signals, endogenous peroxidase activity was blocked by immerging the tissue sections in a 0.3% H2O2 solution for 20 min at room temperature. Only sections for Ki67 immunostaining were exposed to antigen retrieval (heat induced epitope retrieval) by using a 10 mM sodium citrate buffer (pH 6) at 95 °C for 20 min. Moreover, nonspecific binding sites were blocked by using 2.5% normal goat serum for 1 h at room temperature. Then, sections were incubated with the primary antibody for a different amount of time: Ki67 overnight at 4 °C and α-SMA for 1 h at room temperature. After washing three times with PBS, sections were incubated with the secondary antibody (goat anti-mouse biotin-conjugated IgG, 1:200, Dako, Santa Clara, CA, USA) for 30 min at room temperature. Thereafter, an avidin–biotin complex (ABC Reagent, VECTASTAIN^®^ ABC Kit, PK-4000, Vector Laboratories, Burlingame, CA, USA) and 3,3′-diamobenzidine (DAB) system (ImmPACT^®^ DAB Substrate, SK-4105, Vector Laboratories, Burlingame, CA, USA) were used to achieve the immunolabeling. All sections were counterstained with Mayer’s hematoxylin solution. Positive and negative sections controls were always performed in parallel with experimental sections in order to assess the specificity of immunostaining reaction. Immunostained sections were observed under a light microscope provided with a camera (Olympus Vanox AHBT3, Olympus, Tokyo, Japan).

The positive area of Ki67 was quantitatively analyzed. The percentage of positive area was measured in ten randomly selected (400× magnification) fields of view for each slide. The positive signal was measured using an image processing program (ImageJ^®^) and expressed as a percentage of positive area.

α-SMA immunoreactivity was analyzed in a semi-quantitative fashion by scoring the abundancy and orientation of myofibroblasts using a histological score as follows: 0 for absence of immunostaining, 1 for mild presence of immunostaining and irregular pattern, 2 as moderate immunostaining and well-oriented myofibroblasts, and 3 as abundant immunostaining and compact organized myofibroblasts.

### 2.6. Gene Expression Analysis

A total of 24 biopsies, 6 for each time point (3 for treated and 3 for untreated lesions) were used for gene expression analysis by real time PCR (RT-PCR). Total RNA was isolated from skin samples by using TRIzol reagent (Life Technologies, Carlsbad, CA, USA). Then, the RNA extracted was assessed for its quality (260/280 nm wavelengths ratio) and quantified using a Nanodrop spectrophotometer (Thermo Scientific, Waltham, MA, USA). A total amount of 2 µg of RNA was retrotranscribed with Superscript^TM^ II Reverse Transcriptase (Invitrogen, Carlsbad, CA, USA) to obtain complementary DNA (cDNA). Thus, the obtained cDNA was used as template for the RT-PCR gene expression analysis using the ABI 7500 Real-Time PCR system (Applied Biosystems, Foster City, CA, USA). 

The relative expression of genes involved in the skin wound-healing process was evaluated: Collagen 1α1 (Collagen type I, Col1α1), Collagen 3α1 (Collagen type III, Col3α1), vascular endothelial growth factor A (VEGF-A), and hair-Keratin (hKER). The 18S ribosomal RNA (18S) and Ribosomal Protein S24 (RPS24) were used as reference genes in order to normalize the obtained data. Specific pairs of primers for each gene were designed using the Primer Express 3.0 software (Applied Biosystems, Foster City, CA, USA) based on the sheep annotated genome sequence on the GenBank database (sheep genome assembly: GCA_000298735.1) (Supplementary Table S1). Validation and efficiency of the designed primer were assessed by using the standard curve method. All pairs of primers presented an acceptable slope (between −3.8 and −3.3) with a corresponding efficiency of 90–100%. To calculate the efficiency, the ABI 7500 System SDS Software (Applied Biosystems, Foster City, CA, USA) was used.

All experiments were run in triplicate to study the relative gene expression of each gene of interest. A melting curve analysis (dissociation curve) was performed as well to detect the non-specific amplification. The relative expression was calculated by using the 2^−ΔΔCt^ method to normalize the cDNA level of expression of the gene of interest to the reference genes. The uninjured skin was used as the calibrator sample.

### 2.7. Statistical Analysis

All obtained data are expressed as the mean ± standard error of the mean (SEM). All statistical differences were assessed by means of the two-way ANOVA test and statistical differences (*p* ≤ 0.05) were further detected by Dunnett “*post-hoc*” test. Differences in the ETI score were assessed by a Student’s t distribution test and a *p* value less than 0.05 was considered statistically significant. All statistical analyses were performed using the GraphPad Prism 7.0 software (San Diego, CA, USA).

## 3. Results

### 3.1. Clinical Follow-Up 

**Figure 3:**
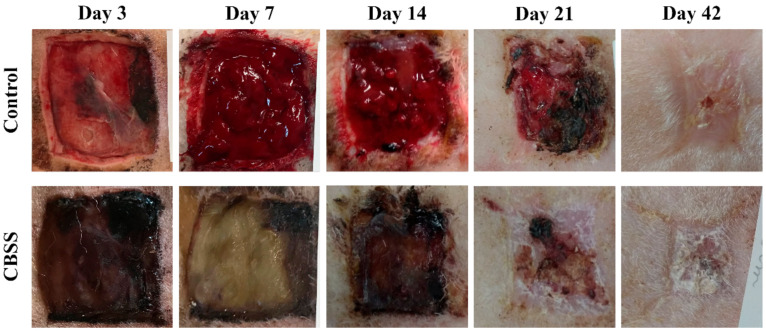
Representative images of the skin ulcers during wound healing.

Wounds treated with the CBSS showed an improved macroscopic quality of the regenerated skin throughout the wound healing process in comparison to untreated (control) wounds. However, neither group reached a complete closure of the wound at 42 days. The presence of the CBSS was observed in treated wounds until day 14 (Figure 3).

One week after wound creation, untreated and CBSS-treated wounds presented a similar percentage of wound contraction (16.250 ± 0.695 vs. 15.313 ± 1.782). At two weeks, CBSS treated wounds showed a slightly higher contraction than the untreated ones (39.188 ± 0.937 vs. 44.708 ± 1.553). Between 21 and 42 days, untreated wounds had a higher percentage of contraction with respect to the CBSS-treated ones (70.917 ± 8.178 vs. 63.583 ± 11.510 at day 21 and 89.333 ± 1.752 vs. 86.896 ± 3.545 at day 42). No significant statistical differences were observed at any time point. 

**Figure 4:**
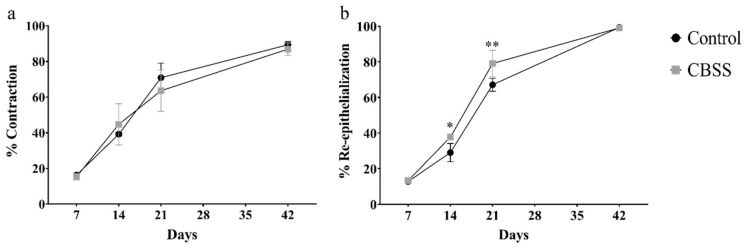
(**a**) Percentage of contraction. (**b**) Percentage of re-epithelialization. Data are shown as mean ± SEM. * *p* < 0.05; ** *p* < 0.01.

At day 7, all wounds showed a similar percentage of re-epithelialization (12.779 ± 1.363 vs. 13.273 ± 0.04). Between 14 and 21 days, CBSS-treated wounds showed a significantly higher percentage of re-epithelialization compared to the untreated wounds: 29.061 ± 5.100 vs. 37.709 ± 0.052 at day 14 (*p* = 0.034) and 67.109 ± 3.667 vs. 79.052 ± 7.398 at day 21 (*p* = 0.0031). At 42 days, all animals presented a similar percentage of re-epithelialization (99.316 ± 1.184 vs. 99.037 ± 1.668) (Figure 4).

### 3.2. Histopathological Analysis

#### 3.2.1. Superficial Inflammation

**Figure 5:**
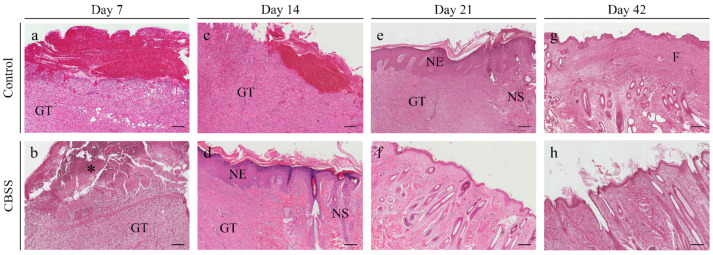
Histopathological microphotographs of skin biopsies at different time points after wounding: control and CBSS-treated wounds comparison. (**a**,**b**) Skin wounds at 7 days, in the CBSS-treated wounds (b) is possible to appreciate the presence of the 3D scaffold; (**c**,**d**) wounds at 14 days, treated wounds started to show a neoepidermis (NE, characterized by an hyperplastic appearance) and skin adnexa; (**e**,**f**) wounds at 21 days after wounding; (**g**,**h**) wounds at 42 days. GT = granulation tissue; NE = neoepidermis; NS = neoskin; F = fibrosis; asterisk = 3D sponge-like scaffold. Scalebar = 200 μm.

At 7 days, the 67% of CBSS-treated wounds showed an abundant inflammatory infiltrate in the superficial region of the dermis while the 33% presented a mild inflammation (Figure 5b). On the contrary, all untreated (control) wounds presented a mild superficial inflammation (Figure 5a). After two weeks, all wounds showed the same amount of inflammation with 67% of the wounds presenting a moderate presence and 33% mild one (Figure 5c,d). At 21 days, CBSS-treated wounds showed a reduction of inflammation with 67% of them presenting a mild inflammation and 33% with no inflammation (Figure 5f). On the contrary, in untreated wounds 67% still showed a moderate amount of inflammation while 33% displayed a mild inflammation (Figure 5e). At 42 days, all wounds showed no evidences of superficial inflammation (100% absent) (Figure 5g,h).

#### 3.2.2. Deep Inflammation

As observed for the superficial inflammation, the deep inflammation parameter showed a decreasing trend throughout the experimentation. At 7 days, 67% of CBSS-treated wounds presented with a moderate inflammation while 33% showed mild inflammation (Figure 5b). At 14 days, in the same group an opposite tendency was observed with 67% of the wounds showing a mild inflammation and 33% a moderate one (Figure 5d). At 21 days, all wounds (100%) showed a mild inflammation in the deeper layer of the skin biopsy (Figure 5f) while at 42 days only 33% presented a mild inflammation, with the remnant 67% without any sign of inflammation (Figure 5h). Untreated wounds showed a mild inflammation (100%) at every time point (Figure 5a–d).

#### 3.2.3. Immature Granulation Tissue

The treatment with the biomaterial led to a higher deposition of granulation tissue than in untreated wounds at 7 days (33% abundant and 67% mild in treated wounds vs. 67% mild and 33% absent in untreated wounds) (Figure 5a,b). At 14 days, a similar amount, but in a different ratio, of immature granulation tissue was observed in treated wounds (67% moderate and 33% mild presence) while all control wounds showed a moderate presence of granulation tissue (100%) (Figure 5c,d). Three weeks post-wounding, 33% of treated wounds showed a moderate amount of immature granulation tissue and 67% a mild one (Figure 5f). Only 33% of untreated wounds showed a mild amount of this parameter and 67% a moderate one (Figure 5e). At 42 days, all wounds did not show any sign of the presence of immature granulation tissue (100% absent) (Figure 5g,h).

#### 3.2.4. Skin Adnexa

Skin adnexa appeared for the first time at 14 days only in CBSS-treated wounds (33% of wounds) (Figure 5d). At 21 days, all treated wounds (100%) showed a moderate amount of skin adnexa while in control wounds only the 33% showed a moderate amount (Figure 5e,f). At 42 days, all treated wounds presented an abundant presence of skin adnexa (Figure 5 h); on the contrary, in untreated wounds 67% presented a moderate amount and 33% an abundant amount (Figure 5g).

#### 3.2.5. Epidermal Thickness Index (ETI)

**Figure 6:**
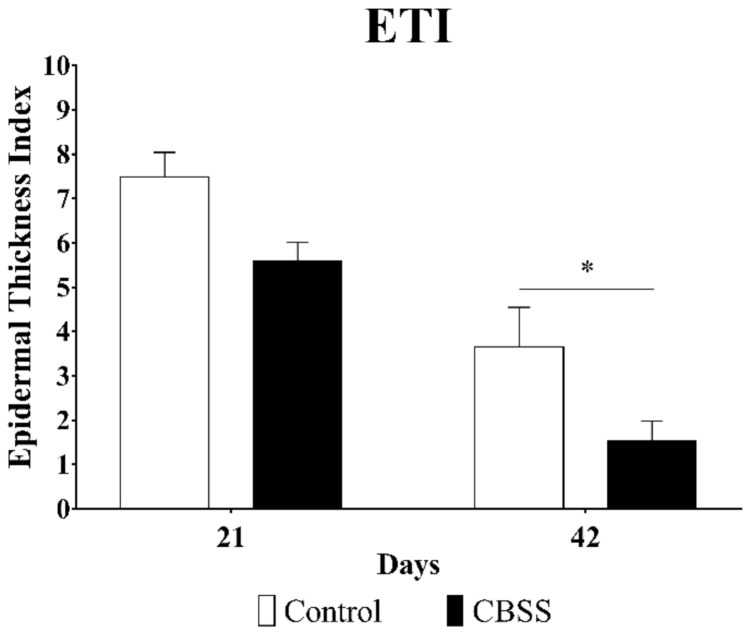
Epidermal thickness index (ETI) at 21 and 42 days respect to unwounded skin. Data are shown as mean ± SEM. * *p* < 0.05.

The control group showed a higher ETI at 21 and 42 days than wounds treated with the CBSS (7.487 ± 0.558 vs. 5.599 ± 0.412 at 21 days and 3.657 ± 0.891 vs. 1.543 ± 0.436 at 42 days). The application of the CBSS led to a reduction of the epidermal thickness at 42 days in a significant way as compared to the control group, more similar to the one of unwounded skin (Figure 6).

#### 3.2.6. Ki67 Immunohistochemistry

**Figure 7:**
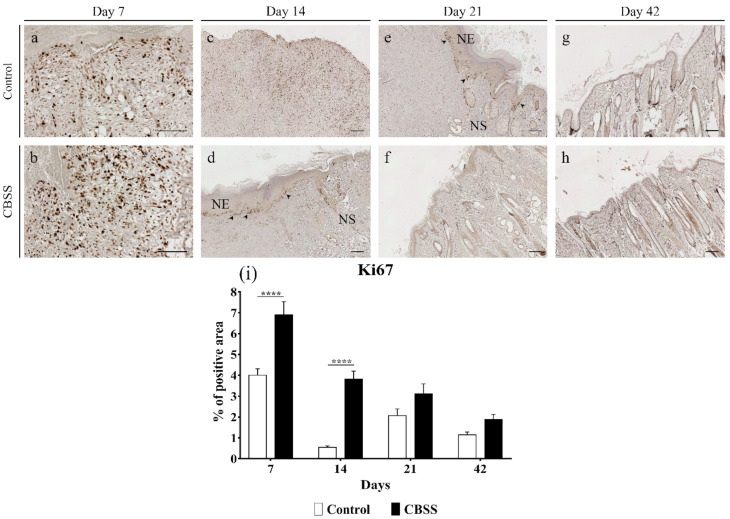
Immunohistochemistry microphotographs for Ki67 immunolabeling. Wounds are showed at (**a**,**b**) 7 days, (**c**,**d**) 14 days, (**e**,**f**) 21 days, and (**g**,**h**) 42 days. (**i**) Quantitative analysis of the percentage of positive area of each sample at 7, 14, 21, and 42 days. Arrowhead = active proliferating keratinocytes in the epidermal basal layer; NE = neoepidermis; NS = neoskin. Scalebar = 200 μm. (**i**) Data are expressed as mean ± SEM. Statistical differences were measured between the two experimental groups at the same time-point. **** *p* < 0.0001.

The immunolabeling for the nuclear protein Ki67, a biological marker for cell proliferation, showed a higher amount of positivity at all time-points in CBSS-treated wounds compared to the untreated wounds, with a statistically significant difference at 7 and 14 days: 4,0080 ± 0.304 vs. 6.902 ± 0.628 at day 7 (*p* < 0.0001), and 0.545 ± 0.072 vs. 3.811 ± 0.390 at day 14 (*p* < 0.0001) (Figure 7a–d,i). The higher amount of Ki67 expression at 14 in CBSS-treated wounds might be attributed to the abundant presence of positivity in the basal layer of the neoepidermis (Figure 7d).

#### 3.2.7. α-SMA Immunohistochemistry

**Figure 8:**
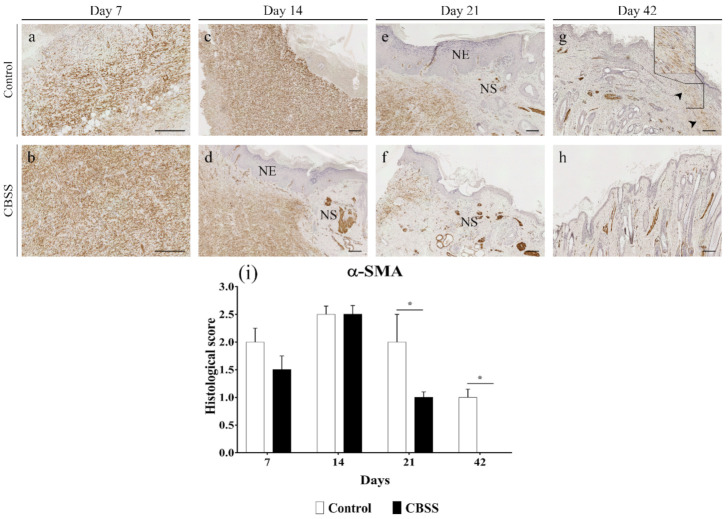
Immunohistochemistry microphotographs for α-SMA immunostaining. Wounds are showed at (**a**,**b**) 7 days, (**c**,**d**) 14 days, (**e**,**f**) 21 days, and (**g**,**h**) 42 days. (**i**) Semi-quantitative analysis based on the score for presence and orientation of myofibroblasts in wounds at 7, 14, 21, and 42 days. Arrowhead = myofibroblasts in the mature dermis; NE = neoepidermis; NS = neoskin. Scalebar = 200 μm; inset = higher magnification of the dermal fibrosis. (**i**) Data are expressed as mean ± SEM. Statistical differences were measured between the two experimental groups at the same time point * *p* < 0.05.

The action of myofibroblasts during skin wound healing is fundamental for wound contraction and deposition of new extracellular matrix components [38]. α-SMA is a reliable myofibroblast marker and was detected since day 7 in all wounds. At 7 and 14 days post-wounding, both treated and untreated wounds showed a similar immunopositive pattern for α-SMA immunostaining. In particular, at 7 days CBSS-treated wounds showed a slightly lower presence of myofibroblasts but better organized (i.e., cells were parallel to each other and to the wound surface) (Figure 8b). At 21 days, the amount of myofibroblasts decreased in wounds treated with the biomaterial while in control wounds the amount of positive cells was similar to day 14; this difference between groups was significant (*p* = 0.0263) (Figure 8e,f). At 42 days, myofibroblasts were still present in untreated wounds (Figure 8g) in the upper layer of the dermis, oriented in parallel to the newly formed epidermis. On the contrary, no myofibroblasts were observed in treated wounds (Figure 8g). It is important to mention that the positivity observed in CBSS-treated wounds at 21 (mainly) and at 42 days has to be ascribed to the myoepithelial cells of apocrine glands and to arrector pili muscles (Figure 8f,h). As a matter of fact, α-SMA is also a marker for smooth muscle cells.

### 3.3. Gene Expression Analysis

**Figure 9:**
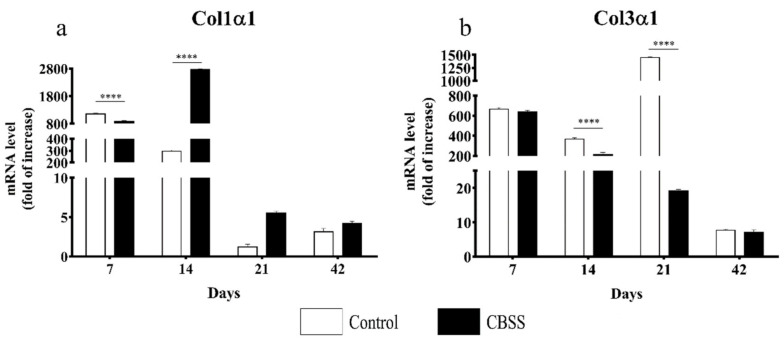
Gene expression analysis for collagen genes involved in skin wound healing. (**a**) Relative expression of the collagen type I (Col1α1) gene and (**b**) collagen type III (Col3α1) gene at 7, 14, 21, and 42 days after wounding in control and CBSS-treated wounds. Data are shown as mean ± SEM. Unwounded skin was used as the calibrator sample. Statistical differences were measured between the two experimental groups at the same time point. **** *p* < 0.0001.

RT-PCR analysis for the gene expression of the two principal collagen types of the skin (I and III) involved in the wound-healing process (in particular, granulation tissue formation and dermis remodeling) revealed an up-regulation of the expression of both genes during the first two weeks after wounding in both groups. At day 7, the gene expression of either type I or III was slightly different between the two groups with a statistically significant difference for collagen type I expression (*p* < 0.0001). However, after two weeks an opposite trend of expression was observed for collagen type I and type III. Indeed, collagen type I gene expression was upregulated in CBSS-treated wounds, while in control group it decreased as compared to day 7, and the difference was statistically different at this time point (*p* < 0.0001). At day 14, even if collagen type III gene expression was lower than day 7 in both groups, it was significantly higher in the control group with respect to the CBSS-treated wounds (*p* < 0.0001). At 21 days, the collagen type I gene expression was reduced for both groups, while the gene expression of collagen type III was strongly upregulated in the control group and decreased in the CBSS-treated group with a statically significant difference (*p* < 0.0001). At 42 days, the gene expression of both genes was similar in the two groups without showing any statistical difference (Figure 9).

**Figure 10:**
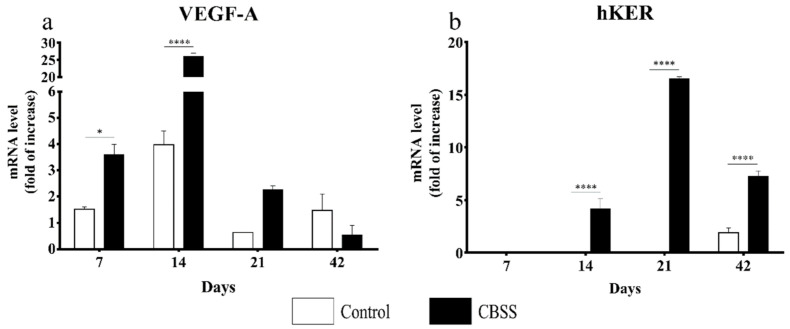
Gene expression analysis for VEGF and hKER genes (**a**) Relative expression of the vascular endothelial growth factor A (VEGF-A) gene and (**b**) hair-Keratin (hKER) gene at 7, 14, 21, and 42 days after wounding in control and CBSS-treated wounds. Data are shown as mean ± SEM. Unwounded skin was used as the calibrator sample. Statistical differences were measured between the two experimental groups at the same time point. * *p* < 0.05; **** *p* < 0.0001.

The gene expression of VEGF-A, an essential growth factor involved in the process of neoangiogenesis, was always higher in the treated wounds in comparison to the untreated ones. In particular, the observed difference of expression was statistically significant at 7 (*p* < 0.05) and 14 days (*p* < 0.0001) (Figure 10a). 

The gene expression of hKER, a marker for the expression of hair follicles, was first detected at day 14 in the CBSS-treated wounds only (Figure 10b). At 21 days, the mRNA level of hKER increased in treated wounds while in the control wounds no expression was observed. At 42 days, the hKER gene expression was lower with respect to day 21 in treated wounds while in untreated wounds it was observed for the first time during the experimental study.

## 4. Discussion

Wound care management of hard-to-heal or chronic ulcers is a critical issue in veterinary medicine since conventional treatments are often associated with a poor prognosis, with a consequent impact on the economic sphere [1,9,10,39,40]. Furthermore, inadequately healing wounds are the second most common cause of euthanasia or death in equine patients [41]. In this context, to overcome this issue, innovative strategies such as tissue engineered skin substitutes have been widely investigated in the recent years [42,43,44]. 

Collagen is the most abundant component of the ECM, therefore a collagen-based biomaterial could be considered as the best option for mimicking the microenvironment of the skin [45]. Collagen derived from marine sources has demonstrated to be a valuable alternative for manufacturing collagenous biomaterials because of its bioactive properties, biocompatibility, and efficiency, as they can stimulate the healing process of the wounded skin [25,46,47]. The collagen used in the present work comes from sea urchin (*P. lividus*) food industry by-products and the herein described collagen-based skin-like scaffold (CBSS) is composed of two layers: the upper one, “epidermal like,” a thin 2D membrane to protect the skin from bacterial infiltration as well to prevent wound dehydration and protein loss, and the lower one, “dermal like,” a 3D porous scaffold that mimics the structure and function of the skin dermis [27]. The cytocompatibility and efficiency of this novel biomaterial has been previously tested in in vitro studies [27,31,32]. The objective of this work was to assess the effects of this innovative bi-layered CBSS on the healing of experimental skin wounds in in vivo sheep model.

Inflammation is a critical step during wound healing. The inflammatory phase is fundamental for clearing the wound area from contaminating pathogens so that the healing process can progress. Nonetheless, an intense and/or prolonged inflammatory response may lead to a delay of healing without further progression to the next phases, hence contributing to wound chronicity [48,49,50]. In the present work, inflammation was examined at the histopathological level. Histopathological findings showed that wounds treated with the CBSS presented a higher presence of inflammatory infiltrate during the first week after wounding compared to wounds left untreated. This scenario might correspond to an anticipated activation of the inflammatory phase because of the biomaterial implantation. One reason underlying this condition could be the earlier activation of platelets by direct interaction with the collagen of the biomaterial [51]. Indeed, it is important to underline that platelets are activated only by collagen in its native fibrillar conformation [52], as the sea urchin-derived collagen used in the CBSS. This property potentially provides these biomaterials also with optimal hemostatic features. After activation, platelets begin to release granules containing several soluble factors; among them, there are different chemotactic molecules (e.g., chemokines, PDGF etc.,) that might have attracted the observed higher amount of inflammatory cells to the wound site during the first week of wound healing with respect to control wounds [53,54,55]. Moreover, collagen-based wound dressings have demonstrated to possess chemotactic properties to inflammatory cells, in particular macrophages [56,57]. However, inflammation diminished throughout the experimentation completely disappearing between 21 and 42 days in treated wounds. On the contrary, it was still mildly present in untreated wounds at day 42 in the deeper layer. The CBSS did not hamper the wound-healing process; on the contrary, it might have anticipated, by inducing it, and shortened the inflammatory phase therefore hastening the progression to the next phase of healing.

Wounds that heal by second intention, like in the current work, are characterized by the formation of granulation tissue: a provisional matrix that covers the wound bed and acts as a scaffold for migrating cells (epithelium, dermis, and vessels). Indeed, the relevant cellularity observed in the granulation tissue is due to the high number of cells proliferating in it [58]. During the first two weeks, CBSS-treated wounds exhibited a higher presence of granulation tissue than untreated wounds, showing how the marine collagen-based biomaterial might have stimulated the production of granulation tissue matrix by fibroblasts. Concomitantly, the higher deposition of granulation tissue in treated wounds might be related to the elevated number of cells in active proliferation observed in the same wounds (Ki67 positive cells). The observed beneficial effects on granulation tissue formation and cells proliferation might be due to the structural characteristics of the CBSS lower layer. Indeed, in our previous work we demonstrated that the 3D sponge-like scaffold was biocompatible plus able to support cell infiltration and proliferation in vitro as it presents peculiar structural characteristics to recreate the dermis in case of injury [27]. Hence, its biocompatibility and structure could have efficiently supported migration, adhesion, and proliferation of fibroblasts in vivo leading to the higher deposition of granulation tissue as observed with other collagenous biomaterials [59,60,61,62,63].

In order to bring nourishment and oxygen to the upper part of the wound, a proper vascularization is vital for the maturation of the granulation tissue, hence for the healing process. Several researchers have proposed skin substitutes containing growth factors and ECM components that could promote wound vascularization [64]. Neovascularization of the injured skin may be the effect of either proliferation of endothelial cells in existing blood vessels (angiogenesis) or differentiation of endothelial progenitor cells (vasculogenesis). Nevertheless, it has been demonstrate that a new vascular network is restored at the wound site, mainly through the mechanism of angiogenesis [65]. Indeed, the pro-angiogenetic activity of CBSS may positively influence the survival of the substitute itself. In the wound model, the CBSS treatment always showed an upregulation of VEGF-A gene expression, a growth factor that supports neoangiogenesis, especially during the first two weeks. Thereby, it could have improved the formation of new vessels and promoted healing through the development of the granulation tissue [47,66,67].

Along with the fibroblasts migrating to the injured site, the keratinocytes surrounding the wound begin to migrate as well to cover it and recreate the epithelial layer [68]. A newly formed epithelium was histologically observed since day 14 in CBSS-treated wounds along with the gene expression of hKER, hence the biomaterial actually accelerated the re-epithelialization process [69,70,71,72]. Indeed, a part of the proliferating cells observed at day 14 was in the basal layer of the epithelium, proving how those cells were actively dividing to recreate a proper epithelial barrier after injury. On the contrary, the epithelium histologically appeared in untreated wounds at day 21 while the gene expression of hKER was detected only after 6 weeks (day 42). 

Besides covering the wound with a new epithelium, the organism exploits another healing mechanism to diminish the open wound area: wound contraction, with wound edges being pulled to the center of the wound by specialized cells [73]. This process is mediated by α-SMA-expressing myofibroblasts, a cell population that differentiate from fibroblasts, and is specialized in secretion of ECM components and granulation tissue contraction [38]. Clinically, the percentage of wound contraction was similar in both groups, but slightly minor in wound treated with the CBSS during the last two weeks. However, the immunohistochemistry staining revealed that the presence of myofibroblasts was always higher in untreated wounds, except at day 14. This observation may be explained by the better parallel organization of myofibroblasts and deposition of collagen fibrils in the treated wounds as compared to untreated wounds. As a matter of fact, for a proper and efficient wound contraction myofibroblasts should be parallelly co-aligned with the collagen fibrils to the wound surface [74], hence increasing the mechanical strength of the tissue and efficiently contracting [75,76].

While contracting, the ECM of the wounded skin begins to remodel itself into a mature dermis with a decreased level of cellularity and proliferation, as well as replacement of the ECM components [77,78]. In the current study, we observed that the skin substitute was able to induce the gene expression of the mature type of collagen, collagen type I, at 14 and 21 days, while in the control wounds it was observed a similar trend but for the collagen type III, the immature type. These differences in levels of expression were reflected in the histological appearance of the skin during the last phases of healing. While treated wounds started to show a compact ECM, a peculiar characteristic of mature dermis, along with the presence of skin appendages (hair follicles and glands), since day 21, the untreated wounds presented a higher ratio of loose ECM (i.e., granulation tissue) until day 42. Collagen-based biomaterials have shown to possess the ability to induce the collagen synthesis [69] and a better organization of collagen fibrils [71]. Apart from starting to express a mature type of collagen earlier than untreated wounds, CBSS application showed a decline in the number of α-SMA positive cells and amount of granulation tissue since day 21, with the complete disappearance of both features by day 42. On the contrary, wounds left untreated still showed an intense positivity for α-SMA protein at day 21 with a moderate presence of granulation tissue. Although the latter was not observed at day 42, α-SMA positive cells were still present in the upper layer of the dermis and organized in a parallel pattern to the wound surface. In addition, histological observations highlighted a high cellularity in the same area with compact collagen fibrils. These two characteristics, plus the upregulation of the gene expression of collagen type III observed at day 21, might be the outcome of an excessive deposition of immature ECM that might have evolved into a pathological fibrotic tissue [79,80]. In particular, it resembles the histological features of a hypertrophic scar, because of the higher thickness of the epidermis with respect to day 0 (epidermal thickness index, ETI, Figure 5) together with parallel myofibroblasts and dense collagen fibrils arrangement [81,82,83,84]. On the contrary, the skin substitute led to a physiological maturation of the granulation tissue with a proper development and organization of the mature dermis (collagen fibrils) and skin appendages. Hence, the treatment reduced the dermal fibrosis, and scarring, in wounds healing by second intention [85,86].

Skin substitutes must possess certain structural characteristics (e.g., biocompatible, control wound contraction, certain porosity for cell infiltration, etc.,) that can provide a support to the wounded skin for the regenerative process [87]; indeed, the CBSS possesses these characteristics as described by Ferrario and colleagues [27]. In addition, the degradation of the biomaterial should be simultaneous to the remodelling of the newly formed dermis in order to sustain its maturation and re-assembling [88,89]. During the experimental period, the presence of the CBSS (upper layer) was evident until day 14 at the macroscopic level (Figure 3). On the contrary, in histological figures (Figure 5), the presence of the CBSS (lower layer) was evident only at day 7 (Figure 5b). After this time point, it was not possible to distinguish between the host dermal collagen and the biomaterial one. As a matter of fact, starting from day 14 we observed a partial regeneration of the neo-dermis, which it might be considered synchronized with the progressive degradation of the biomaterial from our observations [90].

The research in the tissue engineering and regenerative medicine (TERM) field is rapidly evolving and developing new tools for tissue regeneration. The herein presented positive application of a skin substitute (CBSS) might be further improved by combining the marine collagen-based biomaterial with bioactive factors (e.g., growth factors) and/or cells (e.g., mesenchymal stem cells, MSCs). An addition of platelet-rich plasma (PRP), rich in growth factors, might support the healing process by releasing different soluble factors that in combination with skin substitute might limit inflammation, stimulate angiogenesis and re-epithelialization, hence favoring the physiological reparative mechanisms of the skin [91,92]. However, future studies will be necessary to validate the observed results. In addition, a larger number of animals and the comparison of the application of the CBSS with gold standards techniques (skin autograft) or the usage of a commercially available skin substitute will be fundamental to further assess the beneficial outcomes of the CBSS treatment for large skin wounds in the veterinary clinical practice. 

## 5. Conclusions

In this study, the application of an innovative skin substitute made up of collagen derived from marine sources, i.e., sea urchin food wastes, was assessed in an in vivo wound healing model. The skin substitute supported and stimulated wound healing throughout the whole process: it controlled inflammation, promoted the deposition and maturation of granulation tissue, enhanced re-epithelialization, and induced the formation of skin appendages. At the same time, it showed resistance to wound contraction and dermal fibrosis. Overall, the collagen-based biomaterial served as a template for skin wound healing, i.e., as a scaffold for cell migration and growth. All these features led to a better quality of the repaired skin.

## Figures and Tables

**Table 1:** List and details of antibodies used in this study for immunohistochemistry (HIER, heat-induced epitope retrieval; α-SMA, alpha smooth muscle actin).

Antibody	Clonality	Host	Clone	Antigen Retrieval	Dilution	Catalog#
Ki67	Monoclonal	Mouse	MIB-1	HIER, sodium citrate 10 mM pH 6.0	1:50	M7240
α-SMA	Monoclonal	Mouse	1A4	-	1:1000	M0851

## Data Availability

The data presented in this study are available within the article.

## Supplementary Materials

The following are available online at https://www.mdpi.com/article/10.3390/ani11051219/s1. Table S1: List of primer sequences used for the gene expression analysis (RT-PCR).
