# Supplementary material for: A Prototype Skin Substitute, Made of Recycled Marine Collagen, Improves the Skin Regeneration of Sheep
Source: Animals (Basel). 2021 Apr 23;11(5):1219. doi: 10.3390/ani11051219 (PMC8145883; doi:10.3390/ani11051219)
Supplement: Supplementary file 1 [file animals-11-01219-s001.zip › animals-1144062-supplementary/Supplementary materials.pdf]

Table S1. List of primer sequences used for the gene expression analysis (RT-PCR).

Gene	Sequence (5′ → 3′)	Accession number
18S	F: AAACGGCTACCACATCCAAG<br>R: TCCTGTATTGTTATTTTTCGTCAC	XR_003587981.1
Ribosomal protein S24 (RPS24)	F: TTTGCCAGCACCAACGTTG<br>R: AAGGAACGCAAGAACAGAATGAA	XM_004021507.3
Collagen 1α1 (Col1α1)	F: GTACCATGACCGAGACGTGT<br>R: AGATCACGTCATCGCACAGCA	XM_027974707.1
Collagen 3α1 (Col3α1)	F: TCTTCCAATAACACTGCGTTACATTC<br>R: CTAATTTCTTGCTTGACTGTTTCAGAA	XM_004004514.4
Vascular endothelial growth factor A (VEGF-A)	F: GCTCTCTTGGGTGCATTGGA<br>R: TGCAGCCTGGGACCACTT	NM_001025110.1
hair Keratin (hKER)	F: TGGTTCTGTGAGGGCTCCTT<br>R: GGCGCACCTTCTCCAGGTA	NM_001199070.1
